# Indirect Competitive Enzyme-Linked Immunosorbent Assay Based on Broad-Spectrum Antibody for Simultaneous Determination of Thirteen Fluoroquinolone Antibiotics in *Rana catesbeianus*

**DOI:** 10.3390/foods12132530

**Published:** 2023-06-29

**Authors:** Biao Zhang, Yihan Lang, Bowen Guo, Zhengyang Cao, Jin Cheng, Danfeng Cai, Xuping Shentu, Xiaoping Yu

**Affiliations:** Zhejiang Provincial Key Laboratory of Biometrology and Inspection & Quarantine, College of Life Sciences, China Jiliang University, Xueyuan Street, Xiasha Higher Education District, Hangzhou 310018, China; zb@cjlu.edu.cn (B.Z.); langyh@cjlu.edu.cn (Y.L.); p22091055019@cjlu.edu.cn (B.G.); 2200901105@cjlu.edu.cn (Z.C.); 2200901122@cjlu.edu.cn (J.C.); caidanfeng@cjlu.edu.cn (D.C.); yxp@cjlu.edu.cn (X.Y.)

**Keywords:** fluoroquinolones, indirect competitive ELISA, broad-spectrum antibody, *Rana catesbeianus*, multicomponent simultaneous detection

## Abstract

Fluoroquinolone (FQ) is a type of widely used antibiotic in agriculture and aquaculture, and exposure to low doses of FQs may result in the transfer of resistance between animal and human pathogens. Based on the optimization of the operating parameters, an indirect competitive enzyme-linked immunosorbent assay (ic-ELISA) standard curve was constructed for the simultaneous detection of 13 FQs, including enrofloxacin (ENR), ciprofloxacin (CIP), sarafloxacin (SAR), ofloxacin (OFL), norfloxacin (NOR), pefloxacin mesylate (PM), pefloxacin (PEF), enoxacin (ENX), marbofloxacin (MAR), fleroxacin (FLE), lomefloxacin (LOM), danofloxacin (DAN), and difloxacin (DIF). The limit of detection (LOD, computed as IC_10_) and sensitivity (IC_50_) of the ic-ELISA for ENR were 0.59 μg/L and 19.23 μg/L, respectively. The precision and dependability of the detection results of this ic-ELISA were properly verified by HPLC in *Rana catesbeianus* samples. This indicated that the established ic-ELISA approach could be utilized to determine the FQs in *Rana catesbeianus*. In addition, this ic-ELISA, based on a broad-spectrum antibody, provides a technical reference and potential strategy for an immunoassay of hazard factors with similar structure.

## 1. Introduction

Fluoroquinolone (FQ) antibiotics are one of the most commonly used veterinary drugs, with a low cost and high broad-spectrum antibacterial activity against Gram-positive bacteria such as *Staphylococcus aureus* and many others [1,2,3]. However, excessive use breeds bacterial resistance, and FQs’ residues may be transported into the human body through the food chain [4,5,6]. The consumption of animal-derived products that contain excessive amounts of fluoroquinolone for an extended period of time was linked to allergies, hemorrhage, and renal failure, ultimately posing a major risk to human health [7,8,9]. Therefore, FQs are prohibited or restricted in many countries in food-producing animals due to the development of resistance and the impact on human and animal health. Common fluoroquinolones include ciprofloxacin, norfloxacin, ofloxacin, pefloxacin, etc. [10,11]. *Rana catesbeianus* is a sort of popular edible frog, because of its tasty meat and abundant nutrition, with high protein, low fat, and low cholesterol [12,13]. Large-scale farming of *Rana catesbeianus* has rendered it a significant economic breeding animal in China. However, under intensive high-density culture, *Rana catesbeianus* has a slow growth rate, low feed utilization rate, and weak resistance to bacteria and viruses. *Rana catesbeianus* is prone to bacterial or fungal diseases such as red-leg syndrome and chytridiomycosis [14,15]. On account of the pursuit of economic gains, some breeders utilize an excessive amount of prohibited and restricted veterinary drugs during animal feeding and veterinary treatment, which seriously affects the healthy development of the industry and raises social concerns about food safety [16,17]. It is vital to investigate the efficient detection approach of the FQs’ residues in *Rana catesbeianus*, since field testing frequently involves the detection of a large number of samples.

Both domestically and internationally, screening and confirmatory analytical procedures are frequently employed for the detection of veterinary medication residues [18,19]. Due to their remarkable specificity, high accuracy, and outstanding repeatability, liquid chromatography/mass spectrometry (LC/MS) and high-performance liquid chromatography (HPLC) are primarily utilized as confirmatory procedures when analyzing veterinary drug residues in animal-derived foods [20,21,22]. However, these confirmatory methods require tedious pretreatment steps, expensive instruments, specialized technical personnel, and a long testing cycle, which are inappropriate for point-of-care testing (POCT) [23,24,25,26]. In the meantime, due to their rapidity, affordability, mobility, and high sample throughput, screening approaches are increasingly being thought of as alternatives and complementing methods for residue analysis [27,28].

**Figure 1:**
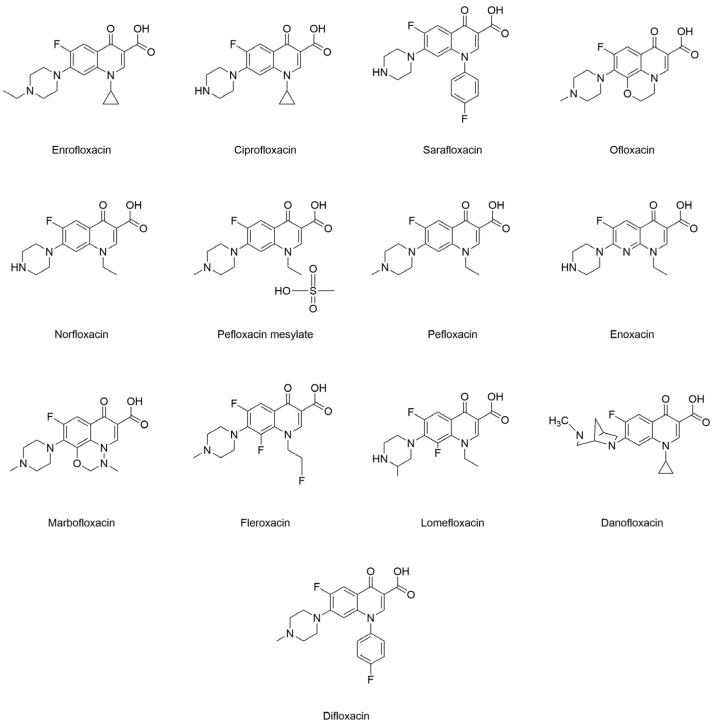
Thirteen FQs’ structural formula.

The advantages of the enzyme-linked immunosorbent assay (ELISA) over other screening techniques include its high sensitivity, minimal instrumentation, and straightforward sample pretreatment [29,30,31]. The kit’s short 2-h analysis period makes it ideal for rapidly recognizing large quantities of samples in the field. There are, currently, studies for determining the residues of FQs in pork, chicken, milk, and animal feeds [32,33,34]. Additionally, an assay utilizing a broad-spectrum-specific antibody to detect multiple FQs’ residues in a single assay is optimal because all FQs have comparable core structures (Figure 1). In this study, we further explored the indirect competitive ELISA (ic-ELISA) approach for the rapid and concurrent detection of 13 FQs’ residues: enrofloxacin (ENR), ciprofloxacin (CIP), sarafloxacin (SAR), ofloxacin (OFL), norfloxacin (NOR). pefloxacin mesylate (PM), pefloxacin (PEF), enoxacin (ENX), marbofloxacin (MAR), fleroxacin (FLE), lomefloxacin (LOM), danofloxacin (DAN), and difloxacin (DIF), with a minimum detection limit of 0.59 μg/L; ic-ELISA was then applied to the detection of an actual aquatic product, *Rana catesbeianus*.

## 2. Materials and Methods

### 2.1. Reagents and Instruments

ENR, CIP, SAR, OFL, NOR, PM, ENX, MAR, FLE, LOM, DAN, DIF, and nalidixic acid were obtained from Sigma-Aldrich (Saint Louis, MO, USA). Malachite green, metronidazole, tetracycline, florfenicol, thiamphenicol, sulfamethazine, sulfamethoxazole, and Tween-20 were obtained from Macklin Biochemical Co., Ltd. (Shanghai, China). PEF, 3,3′,5,5′-Tetramethylbenzidine (TMB), dimethyl sulfoxide (DMSO), sodium carbonate (Na_2_CO_3_), sodium bicarbonate (NaHCO_3_), sodium chloride (NaCl), skimmed milk powder, acetonitrile, sodium hydroxide (NaOH), dichloromethane, n-hexane, phosphoric acid, and methanol were obtained from Aladdin Reagent Co., Ltd. (Shanghai, China). Goat anti-mouse HRP was obtained from Jackson Immunoresearch Laboratories (West Grove, PA, USA). Chloramphenicol was obtained from Dr. Ehrenstorfer GmbH (Augsburg, Germany). Anti-OFL monoclonal antibody, ENR coating antigen was purchased from Landu Biotechnology Co., Ltd. (Binzhou, China). A phosphate buffer saline (PBS buffer, 0.01 mol/L, pH 7.4) was obtained by accurately weighing 9.00 g NaCl, 13.76 g Na_2_HPO_4_·12H_2_O, and 1.79 g NaH_2_PO4·2H_2_O and then adding ultra-pure water to 1000 mL. Phosphate buffer solution (PBST) was obtained from 995 mL PBS buffer (10 mM, pH 7.4) containing 10% Tween-20.

Ninety-six-well EIA/RIA plates were obtained from Corning Incorporated (New York, NY, USA). Varioskan LUX multimode microplate reader was obtained from Thermo Fisher Scientific Co. (Waltham, MA, USA) to measure the absorbance. XTerra MS C_18_ column and Arc HPLC System with 2998 PDA Detector were obtained from Waters (Shanghai, China). Rotary evaporator was obtained from BUCHI Corporation (New Castle, DE, USA). Dry nitrogen blower was obtained from Qiuzuo Scientific Instrument Co., LTD. (Shanghai, China). Milli-Q water (Millipore, MA, USA) was used in all the experiments.

### 2.2. Development of ic-ELISA

With the intention of progressing this supplied ic-ELISA, the critical experimental parametric settings, such as the concentration of coated antigen and dilution factor of antibody were modified. In order to determine the ideal ic-ELISA conditions, different concentrations of coated antigen (0.05, 0.1, 0.2 μg/well) and a series of dilution coefficient of antibody (1:32,000, 1:64,000, 1:128,000, 1:256,000, 1:512,000) were selected by chessboard titration. The ic-ELISA approach could be described as follows: A 96-well plate with the coating antigen solution (100 µL/well) added was incubated at 4 °C overnight. The microwells were blocked through the addition of the blocking solution (200 µL/well) (0.5% skimmed milk powder solution) at 37 °C for 1 h, after being washed three times with PBST (0.01 mol/L PBS with 10% Tween-20). Following the cleaning of the microwells, FQs’ standard or sample solution (50 µL/well) and antibody in PBS (50 µL/well) were added to each well. The microwell plate was then incubated at 37 °C for 1 h. After washing, the 500-fold HRP-labeled secondary antibody solution (100 µL/well) was added to each well, and then the microwell plate was incubated at 37 °C for 30 min. Following cleaning again, TMB substrate solution (100 µL/well) was applied to microwells and then incubated for 15 min at 37 °C. The injection of 50 µL H_2_SO_4_ (1.25 mol/L) halted the reaction in each well. The absorbance was eventually determined in a multimode microplate reader utilizing a 450 nm test wavelength [35].

### 2.3. Determination of Sensitivity and Specificity

Sensitivity and specificity are crucial parameters in evaluating ELISA techniques. To determine the inhibition rate (%), several FQs’ standard solution concentrations were utilized (0.01, 0.05, 0.5, 1, 5, 10, 50, 100, 500, 2000, 10,000 μg/L). The following is the formula for estimating the inhibition rate:

$$\mathrm{inhibition}\;\mathrm{rate}\left(\%\right)=\left(A-A_{i}\right)/\left(A-A_{0}\right)\times 100$$

where, *A*_0_ is the absorbance value of well with 100 μL PBS solution added, *A_i_* is the absorbance value of the well with 50 μL antibody and 50 μL different doses of FQs’ solution added, and *A* is the absorbance value (at 450 nm) of the well with 50 μL antibody and 50 μL PBS solution added, respectively. An ic-ELISA approach was brought by utilizing the optimal working parameters and gradient concentration standards (0.01, 0.05, 0.5, 1, 5, 10, 50, 100, 500, 2000, 10,000 μg/L). The four-parameter equation was fitted with the average of three distinct experiments performed in triplicate, as reported in Supplementary Table S1. The logistic 5 fitting equation in Origin pro 2021 software was applied to calculate the sensitivity (50% inhibitory concentration, computed as IC_50_), as well as the limit of detection (LOD, computed as IC_10_).

Specificity was defined as the capacity of structurally or functionally related compounds to bind to the antibody, and cross-reactivity (CR) was calculated as:

$$\mathrm{Crossreactivity}\;\left(\%\right)=100\times {\mathrm{IC}}_{50}\left(\mathrm{ENR}\right)/{\mathrm{IC}}_{50}\left(\mathrm{other}\;\mathrm{analogues}\right)$$

In the current investigation, CIP, SAR, OFL, NOR, PM, PEF, ENX, MAR, FLE, LOM, DAN, DIF, and nalidixic acid, which were similar to the structure of ENR, and additional 8 common veterinary antibiotics for aquatic products (including malachite green, metronidazole, tetracycline, florfenicol, thiamphenicol, chloramphenicol, sulfamethazine, sulfamethoxazole) were selected for specificity determination. The ratio of IC_50_ of ENR to IC_50_ of other antibiotics was utilized as the cross-reactivity.

### 2.4. Sample Preparation

Samples of commercial *Rana catesbeianus* were acquired from nearby supermarkets in Hangzhou, Zhejiang, China. Prior to application, all samples were analyzed to ensure they were free of FQs. For edible animal tissues, such as porcine muscle, chicken muscle, fish, shrimp, porcine liver, chicken liver, and other organs, organic solvent extraction was typically utilized. The sample pretreatment method was modified according to the Ministry of China Agriculture Announcement No. 1025-8-2008 [36]: 4.00 g sample and 12 mL acetonitrile-0.1 mol/L sodium hydroxide solution was added into a 50 mL centrifuge tube, shaken for 5 min, and centrifuged at 4000 r/min for 5 min. Then, 6 mL of supernatant was taken, and 6 mL of 0.02 mol/L phosphate buffer solution and 7 mL of dichloromethane was added and then shaken for 5 min. The lower organic phase (6 mL) was dried by nitrogen at 50 °C in a 10 mL glass test tube after centrifugating at 4000 r/min for 5 min. Next, 100 μL of subnatant liquid was removed for further analysis after adding 1 mL of 0.02 mol/L phosphate buffer, eddying for 2 min, then adding 2 mL of n-hexane, and eddying for 30 s. For the purpose of reducing matrix interference, the extracts were redissolved with PBS buffer. The supernatant was diluted by 2 times, 4 times, 6 times, 8 times, and 10 times. By comparing the established matrix curve and standard curve, the dilution multiple, which basically eliminated the matrix effect, was selected for follow-up ic-ELISA experiments.

### 2.5. Assay Validation

It is vital to confirm the objectivity, accuracy, and feasibility of the established analysis approach, with the goal to demonstrate that it is capable of serving the purpose and needs of detection to evaluate the validity and precision of the supplied ic-ELISA. In this research, negative samples of *Rana catesbeianus* without ENR were spiked with various ENR concentrations (50, 100, 200 μg/kg) and concurrently assessed by HPLC and ic-ELISA. The *Rana catesbeianus* samples that contained PEF, DAN, MAR, FLE, OFL, NOR, CIP, LOM, and ENR (100 μg/kg each) were pitched with the purpose of determining the aggregate amount of FQs by the established ic-ELISA, with the aim of further evaluating the viability of the approach. HPLC was employed to verify the correctness of the results in samples by applying the Chinese National Standard GB 31656.3-2021 with a few modifications [37]. For the chromatographic separation, the XTerra MS C_18_ column (4.6 mm × 250 mm, 5 μm) was utilized. In this research, the Arc HPLC System with 2998 PDA Detector (ultraviolet detector) was utilized, the detection wavelength was 278 nm, the column temperature was 30 °C, the flow rate was 0.8 mL/min, and the injection volume was 10 μL The mobile phases were as follows: A: 0.3% phosphoric acid-5% acetonitrile aqueous solution (pH 4.00 ± 0.01 adjusted with triethylamine); B: methanol [38]. The detecting condition of linear gradient for HPLC analysis was as follows: the volume of mobile phase B was 15% at 0–5 min, increased linearly to 40% at 5–45 min, and then returned to 15% at 45–50 min. The linear gradient parameters for HPLC analysis are reported in Table 1. The sample processing steps are listed in detail in the supporting information.

## 3. Results and Discussion

### 3.1. Optimization of Working Parameters

**Figure 2:**
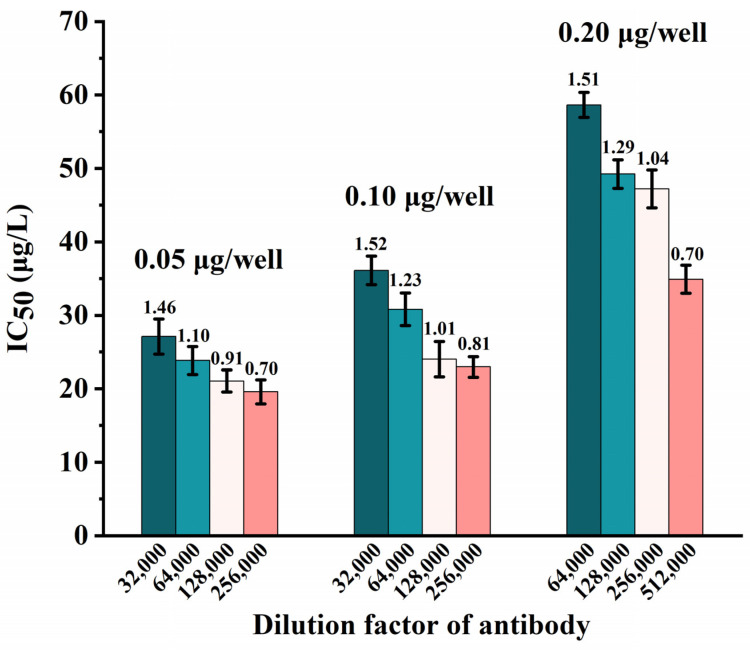
Optimization of antibody dilution (1:32,000, 1:64,000, 1:128,000, 1:256,000, 1:512,000) and coating antigen concentration (0.05, 0.10, 0.20 μg/well) optimization. Each corresponding column bears a mark indicating the average OD value.

The lowest IC_50_ value with a suitable OD value (ranging from 0.8 to 1.2) is necessary to construct a sensitive ELISA assay. Massive errors may result from OD values that are either too high or too low, which affect the detection sensitivity of the ic-ELISA. In order to achieve the ideal outcomes, the following variables were optimized: the dilution coefficient of the antibody (1:32,000, 1:64,000, 1:128,000, 1:256,000, 1:512,000) and the amount of the coated antigen (0.05, 0.1, 0.2 μg/well). The results are shown in Figure 2. The maximum absorbance (OD_max_), which indicates the maximum binding between the antibody and coated antigen, was 1.46 when the coating antigen’s concentration was 0.05 μg/well and the dilution coefficient of the antibody was 1:32,000, which was beyond the range of the optimal OD value. The OD_max_ value was 0.70 when the dilution coefficient of the antibody was 1:256,000, and the lower OD value would cause the instrument reading to be inaccurate. The detection sensitivity (IC_50_) was 27.11, 23.83, 21.05, and 19.58 μg/L, when the coating antigen concentration was 0.05 μg/well and the dilution coefficient of the antibody was 1:32,000, 1:64,000, 1:128,000, and 1:256,000, respectively. When the coating antigen concentration was 0.10 μg/well, the half-maximal inhibitory concentration was 36.11, 30.80, 24.03, and 22.97 μg/L, when the dilution coefficient of the antibody was 1:32,000, 1:64,000, 1:128,000, and 1:256,000, respectively. When the coating antigen concentration was 0.20 μg/well, the half-maximal inhibitory concentration was 58.65, 49.24, 47.21, and 34.90 μg/L, when the dilution coefficient of the antibody was 1:64,000, 1:128,000, 1:256,000, and 1:512,000, respectively. The detection sensitivity was not as excellent as 0.05 μg/well when the coated antigen was 0.10 μg/well and 0.20 μg/well; hence, the study did not adopt these amounts. The experimental effect was optimum when the amount of the coated antigen was 0.05 μg/well and the dilution coefficient of the antibody was 1:128,000, since this resulted in an OD_max_ value of 0.91 and a half-maximal inhibitory concentration of 21.09 μg/L. The fitting curve tended to be smooth and a typical S-shaped curve. The OD_max_ value of the operating parameters had high sensitivity and was appropriate for ELISA detection.

### 3.2. Establishment of icELISA Standard Curve

**Figure 3:**
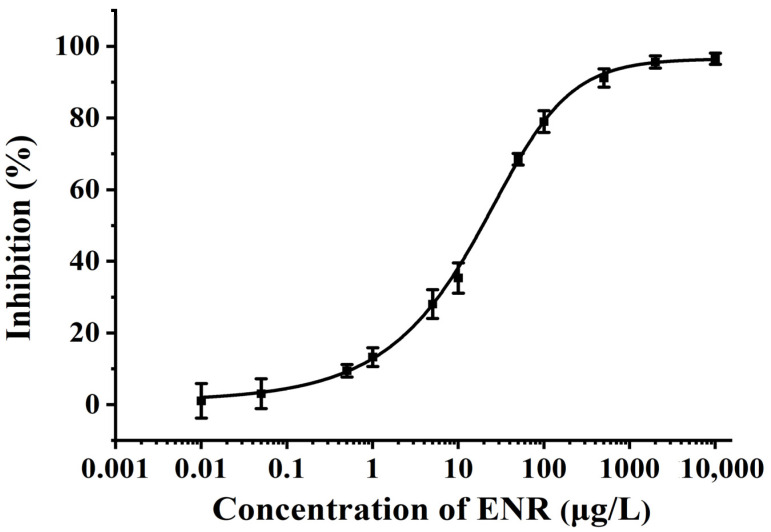
Standard inhibition curve of ic-ELISA for detecting ENR in PBS.

The IC_10_ values and IC_50_ values were derived from the standard curves, which were employed to evaluate the detection limit and detection sensitivity of the generated ic-ELISA. Under the above-mentioned optimum operating parameters, the standard curves were obtained from various concentrations of FQs’ standard solution as the abscissa (0.01, 0.05, 0.5, 1,5, 10, 50, 100, 500, 2000, 10,000 μg/L) and the inhibition rate as the ordinate. The standard inhibition curves of 13 FQs (ENR, CIP, SAR, OFL, NOR, PM, PEF, ENX, MAR, FLE, LOM, DAN, DIF) in PBS by the ic-ELISA were fitted by Origin. The standard curve of ENR is displayed in Figure 3 (R^2^ = 0.9995), and the standard curves of CIP, SAR, OFL, NOR, PM, PEF, ENX, MAR, FLE, LOM, DAN, and DIF are shown in Supplementary Figure S1 (R^2^ = 0.9984, 0.9964, 0.9986, 0.9923, 0.9994, 0.9991, 0.9990, 0.9976, 0.9949, 0.9981, 0.9974, 0.9978), all of which were typical S-shaped curves. The concentration of FQs in the aforementioned 13 fitting curves all remained stable when they were less than 0.01 μg/L or more than 10,000 μg/L. Utilizing this means, the detection limits (IC_10_) of ENR, CIP, SAR, OFL, NOR, PM, PEF, ENX, MAR, FLE, LOM, DAN, and DIF were 0.59, 0.97, 1.02, 1.15, 1.37, 1.38, 1.44, 1.66, 1.86, 2.01, 2.04, 2.71, and 3.61 μg/L, respectively, and the half-maximal inhibitory concentrations (IC_50_) were 19.23, 18.69, 21.55, 22.52, 21.84, 20.93, 21.96, 21.84, 21.69, 21.90, 22.35, 20.61, and 22.52 μg/L, respectively, as reported in Supplementary Table S2. The Ministry of China Agriculture Announcement No. 1025-8-2008 detected ENR, CIP, NOR, OFL, LOM, oxaquic acid, ENX, PEF, DAN, flumequine, MAR, and amifloxacin residues in pig muscle, chicken muscle, chicken liver, honey, eggs, and shrimp, in foods of animal origin, with a detection limit of 3 μg/kg. NOR, CIP, ENR, OFL, oxaquic acid, and flumequine were found in the edible tissues of fish, shrimp, crab, and shellfish, with a detection limit of 2.5 μg/kg, according to the Chinese National Standard GB 31656.3-2021. Due to the lack of antibodies with the appropriate affinity and specificity, previous immunoassay approaches were typically restricted to the detection of a single FQ type or a small number of FQ types. The detection limit of the ic-ELISA, based on a monoclonal antibody against LOM employed by Mukunzi et al. to identify LOM and related drug residues in milk, was 0.04 ng/mL in 0.1 M phosphate-buffered saline (PBS) [39]. The method was highly sensitive to LOM, NOR, and ENX, moderately sensitive to CIP and OFL, and slightly sensitive to FLE and PEF, with a CR < 0.1% compared to the other 12 FQs.

### 3.3. Determination of Specificity

All immunoassays have the inherent property of specificity. The specificity of the established ic-ELISA was evaluated by calculating the ratio of the IC_50_ of ENR to the IC_50_ of other veterinary antibiotics. In this study, we sought to establish an ic-ELISA method capable of recognizing the majority of common FQ analogs. The IC_50_ values of FQs (CIP, SAR, OFL, NOR, PM, PEF, ENX, MAR, FLE, LOM, DAN, DIF, nalidixic acid) with a similar structure to ENR and an additional eight common veterinary antibiotics (malachite green, fungicide, cephalosporin, florfenicol, thiamphenicol, chloramphenicol, sulfamethazine, sulfamethoxazole) utilized in aquatic products were determined by an ic-ELISA based on an anti-OFL monoclonal antibody and an ENR coating antigen. The results presented in Table 2 demonstrated that the recognition capacity of this ic-ELISA to other antibiotic veterinary drugs was investigated. Table 2 lists the half-maximal inhibitory concentration for the 12 FQs’ analogs, including CIP, SAR, OFL, NOR, PM, PEF, ENX, MAR, FLE, LOM, DAN, and DIF, which were 19.23, 18.69, 21.55, 22.52, 21.84, 20.93, 21.96, 21.84, 21.69, 21.90, 22.35, 20.61, and 22.52 μg/L, respectively. Therefore, the cross-reactivities (%) of CIP, SAR, OFL, NOR, PM, PEF, ENX, MAR, FLE, LOM, DAN, and DIF were 102.88%, 89.24%, 85.41%, 88.06%, 91.88%, 87.58%, 88.07%, 88.64%, 87.81%, 86.06%, 93.31%, and 85.38%, respectively. As we expected, these cross-reactivities (%) were all higher than 85%, demonstrating that CIP, SAR, OFL, NOR, PM, PEF, ENX, MAR, FLE, LOM, DAN, and DIF could be recognized by utilizing the established ELISA approach. Meanwhile, the cross-reactivity (%) of this ic-ELISA was 14.97% for nalidixic acid. In addition, the findings demonstrated that the IC_50_ of the above eight antibiotic veterinary drugs was greater than 15,000 μg/L, and the cross-reactivity (%) was less than 0.2%, indicating that the ic-ELISA had no obvious recognition ability for them. As a result, the established ic-ELISA approach has broad-spectrum specificity for the 13 FQs, with low cross-reactivity with nalidixic acid (<15%), and other antibiotic veterinary medications have no impact on the properties of the ic-ELISA (<0.2%).

### 3.4. Removal of Matrix Effects

**Figure 4:**
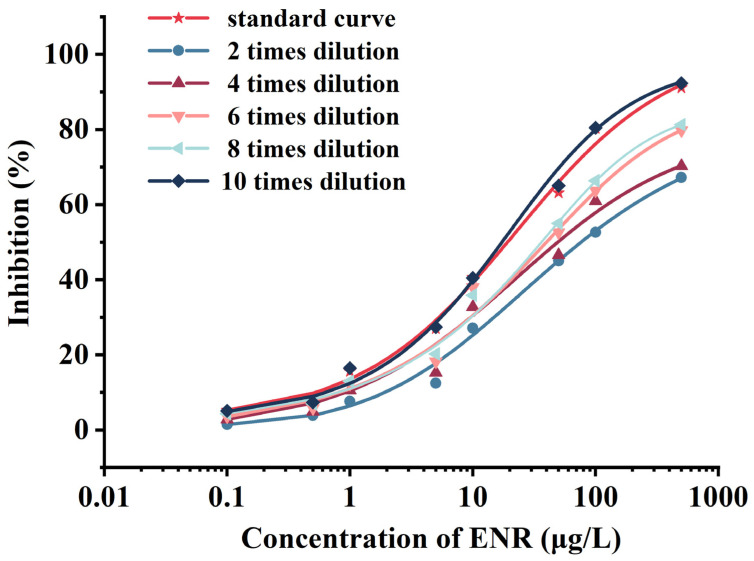
Matrix effect elimination curve of *Rana catesbeianus*.

In general, the recovery rate may be affected by the sample type, analyte solubility, sample preparation technique, and antibody effectiveness. In the actual analysis of food samples, compounds such as proteins and fats in complex food matrices may reduce the intensity of color development or interfere with the binding of antibodies and analytes, thus reducing the accuracy of the results. Therefore, the removal of matrix effects is primary in ELISA experiments. Typically, diluting sample extracts and preparing standard curves in blank tissue are two effective methods for minimizing matrix interference. After the sample extracts of *Rana catesbeianus* were pretreated with the modified Ministry of China Agriculture Announcement No. 1025-8-2008, the extracts were redissolved with PBS buffer. Following redissolving, the extracts were diluted with PBS by 2-fold, 4-fold, 6-fold, 8-fold, and 10-fold, and the matrix curve was generated. The matrix curve was compared with the standard curve obtained in the blank tissue. As shown in Figure 4, when the sample extract was diluted by 2-fold, 4-fold, 6-fold, and 8-fold, then the matrix curve of the *Rana catesbeianus* extract sample significantly deviated from the standard curve, indicating that the matrix effect was still clearly visible; when the extract was diluted 10-fold, the matrix curve was basically consistent with the standard curve, and the matrix effect was negligible. As a consequence, this investigation utilized the 10-fold diluted *Rana catesbeianus* extract sample for subsequent ic-ELISA detection.

### 3.5. Analysis of Spiked Samples

The recoveries of ENR in *Rana catesbeianus* samples were employed by HPLC. Three various spiking concentrations of ENR (50, 100, 200 μg/kg) were set to confirm the viability of the established ELISA approach. The unspiked samples yielded no positive results throughout the spiking and recovery testing, which confirmed that the ELISA method had a low false positive rate and was more suitable for rapid detection in the actual field. *Rana catesbeianus* spiked with FQs exhibited an excellent correlation between the spiking degree and the observed concentration. The results of recovery are shown in Table 3. It can be seen from Table 3 that when the spiking concentrations were 50, 100, and 200 μg/kg, the recoveries of ENR in *Rana catesbeianus* samples by the ic-ELISA were 93.87%, 90.39%, and 90.89%, respectively, while those by HPLC were 85.88%, 102.70%, and 92.18%, respectively. The coefficients of variation (CVs) of the ic-ELISA were 3.86%, 2.30%, and 5.11%, and those of HPLC were 7.01%, 3.18%, and 1.52%, respectively. The CVs of both approaches were less than 10%. The ic-ELISA outcomes were consistent with those of HPLC with a correlation coefficient (R^2^) of 0.9936. For the analysis of the ENR residues in *Rana catesbeianus*, the sample extraction technique and ic-ELISA were satisfactory.

Moreover, since multiple FQs may be simultaneously present in the samples, we measured the concentrations of nine FQs (ENR, CIP, OFL, NOR, PEF, MAR, FLE, LOM, DAN) in commercial *Rana catesbeianus* samples by the ic-ELISA and HPLC to calculate the feasibility and practicality of the existing ic-ELISA. According to Table 4, the concentrations of ENR, CIP, OFL, NOR, PEF, MAR, FLE, LOM, and DAN in *Rana catesbeianus* evaluated by HPLC were 105.83, 118.30, 106.76, 106.18, 71.36, 96.93, 103.76, 94.37, and 94.11 μg/kg, respectively, and the CVs were 7.73%, 2.12%, 1.75%, 0.81%, 9.66%, 1.88%, 0.78%, 9.69%, and 2.87%, respectively. The overall contents of the aforementioned FQs were 897.60 μg/kg, and the CVs were all less than 10%, which was essentially consistent with the total concentration of the nine FQs measured by the ic-ELISA method (782.91 μg/kg) proposed in this paper. The CV of the above ic-ELISA was 8.53%, which proved that the ic-ELISA and HPLC had high accuracy. We found that the accuracy of both the ic-ELISA and HPLC results in determining the concentration of FQs in mixed samples was impacted by the competition amongst the FQs. The total concentration determined by HPLC was higher than the actual spiking concentration, and the total concentration determined by the ic-ELISA was lower than the actual spiking concentration, though still within the controllable range. These discoveries revealed that the ic-ELISA could be utilized to exactly and numerically identify the total amount of ENR, CIP, OFL, NOR, PM, PEF, MAR, FLE, LOM, and DAN in *Rana catesbeianus* samples.

## 4. Conclusions

In summary, the ic-ELISA was established employing broad-spectrum-specific antibodies with comparable recognition ability for 13 FQs, including ENR, CIP, SAR, OFL, NOR, PM, PEF, ENX, MAR, FLE, LOM, DAN, and DIF. Concurrently, the approach had a cross-reactivity of less than 0.2% when detecting the other eight antibiotic veterinary drugs and was moderately sensitive to nalidixic acid. The optimized ic-ELISA demonstrated the LOD of 0.59 μg/L for ENR in PBS. The half-maximal inhibitory concentration (IC_50_) was 19.23 μg/L and had significant cross-reactivity values (>85.38%) for the other 12 FQs (CIP, SAR, OFL, NOR, PM, PEF, ENX, MAR, FLE, LOM, DAN, DIF). It was verified that the matrix effect was negligible with a 10-fold dilution of the optimized *Rana catesbeianus* extract samples. The outcomes of the proposed ic-ELISA were compared to those of HPLC, which showed a strong agreement between them (R^2^ = 0.9936). The total content of the nine FQs in *Rana catesbeianus* samples that were commercially accessible was effectively determined by utilizing the ic-ELISA. In conclusion, the created ic-ELISA could potentially be employed in a rapid, precise, and affordable manner to screen for the drug residues of the FQs in *Rana catesbeianus*.

## Figures and Tables

**Table 1:** Detecting condition of linear gradient for HPLC analysis.

Time (min)	A: 0.3% Phosphoric Acid-5% Acetonitrile Aqueous Solution (%)	B: Methanol (%)
0	85	15
5	85	15
45	60	40
50	85	15

**Table 2:** Specificity evaluation of ic-ELISA (*n* = 3).

Name	IC_50_ (μg/L)	Cross-Reactivity (%)
ENR	19.23	100.00
CIP	18.69	102.88
SAR	21.55	89.24
OFL	22.52	85.41
NOR	21.84	88.06
PM	20.93	91.88
PEF	21.96	87.58
ENX	21.84	88.07
MAR	21.69	88.64
FLE	21.90	87.81
LOM	22.35	86.06
DAN	20.61	93.31
DIF	22.52	85.38
nalidixic acid	128.43	14.97
malachite green	>15,000	<0.2
metronidazole	>15,000	<0.2
tetracycline	>15,000	<0.2
florfenicol	>15,000	<0.2
thiamphenicol	>15,000	<0.2
chloramphenicol	>15,000	<0.2
sulfamethazine	>15,000	<0.2
sulfamethoxazole	>15,000	<0.2

**Table 3:** Recoveries of ENR in *Rana catesbeianus* by ic-ELISA and HPLC (*n* = 3).

Sample	Spiked Concn (μg/kg)	ic-ELISA			HPLC		
		Detected (Mean ± SD ^a^) (μg/kg)	Recovery (%)	CV (%) ^b^	Detected (Mean ± SD) (μg/kg)	Recovery (%)	CV (%)
*Rana catesbeianus*	0	ND ^c^	−	−	ND	−	−
	50	46.94 ± 1.81	93.87	3.86	42.94 ± 3.01	85.88	7.01
	100	90.39 ± 2.08	90.39	2.30	102.70 ± 3.27	102.70	3.18
	200	181.78 ± 9.29	90.89	5.11	184.35 ± 2.81	92.18	1.52

^a^ SD, standard deviation. ^b^ CV, coefficient of variation. ^c^ ND, not detected.

**Table 4:** Determination of 9 FQs in *Rana catesbeianus* utilizing HPLC and ic-ELISA (*n* = 3).

Sample	FQs	HPLC			ic-ELISA	
		Detected (Mean ± SD ^a^) (μg/kg)	CV ^b^ (%)	Total (μg/kg)	Detected (Mean ± SD) (μg/kg)	CV (%)
*Rana catesbeianus*	PEF	71.36 ± 6.89	9.66	897.60	782.91 ± 66.78	8.53
	DAN	94.11 ± 2.70	2.87			
	MAR	96.93 ± 1.82	1.88			
	FLE	103.76 ± 0.81	0.78			
	OFL	106.76 ± 1.87	1.75			
	NOR	106.18 ± 0.86	0.81			
	CIP	118.30 ± 2.51	2.12			
	LOM	94.37 ± 9.14	9.69			
	ENR	105.83 ± 8.18	7.73			

^a^ SD, standard deviation. ^b^ CV, coefficient of variation.

## Data Availability

The datasets generated for this study are available on request to the corresponding author.

## Supplementary Materials

The following supporting information can be downloaded at https://www.mdpi.com/article/10.3390/foods12132530/s1. Experimental methods, Figure S1: Standard inhibition curve of ic-ELISA for detecting 12 FQs (CIP, SAR, OFL, NOR, PM, PEF, ENX, MAR, FLE, LOM, DAN, DIF) in PBS; Table S1: Four-parameter equation of ic-ELISA for detecting 13 FQs (ENR, CIP, SAR, OFL, NOR, PM, PEF, ENX, MAR, FLE, LOM, DAN, DIF) in PBS; Table S2: Detection limits (IC_10_) and half-maximal inhibitory concentration (IC_50_) of ic-ELISA for detecting 13 FQs.
