# Supplementary material for: Indirect Competitive Enzyme-Linked Immunosorbent Assay Based on Broad-Spectrum Antibody for Simultaneous Determination of Thirteen Fluoroquinolone Antibiotics in Rana catesbeianus
Source: Foods. 2023 Jun 29;12(13):2530. doi: 10.3390/foods12132530 (PMC10340495; doi:10.3390/foods12132530)
Supplement: Supplementary file 1 [file foods-12-02530-s001.zip › foods-2451444-supplementary.pdf]

Indirect Competitive Enzyme-Linked Immunosorbent Assay Based on Broad-Spectrum Antibody for Simultaneous Determination of Thirteen Fluoroquinolone Antibiotics in *Rana catesbeianus*

Biao Zhang †, Yihan Lang †, Bowen Guo, Zhengyang Cao, Jin Cheng, Danfeng Cai, Xuping Shentu * and Xiaoping Yu

Zhejiang Provincial Key Laboratory of Biometrology and Inspection & Quarantine, College of Life Sciences, China Jiliang University, Xueyuan Street, Xiasha Higher Education District, Hangzhou 310018, China

* Correspondence: stxp@cjlu.edu.cn

† These authors contributed equally to this work.

Contents

Supplementary experimental method

Table S1 Table S2

Figure S1

Sample Treatment Procedure for HPLC

1. **Extraction**

5.00 g of sample was weighed, 20 mL of acidified acetonitrile solution and 10 g of anhydrous sodium sulfate were added, homogenized at high speed for 1-2 min, centrifuged at 3000 r/min for 5 min, and the supernatant was taken to the funnel. 20 mL of acidified acetonitrile solution was used to clean the cutting head and dissolve the precipitate. The extraction was repeated twice and the supernatant was combined. 60 mL of acetonitrile saturated n-hexane solution was added, and the solution was shaken on the separator funnel shaker (150 r/min) for 20 min. After sitting for a while, the lower acetonitrile was taken to the pear-shaped bottle and evaporated to dryness at 40 °C. After adding 4 mL of phosphate buffer, ultrasonically oscillating for 1 min, and standing for 30 s, the solution was transferred to a 15 mL centrifuge tube. The residue was extracted twice again, and the solution was transferred to the same centrifuge tube. The residue solution was combined 3 times, centrifuged at 3000 r/min for 5 min, and the supernatant was taken for later use.

2. **Purification**

The C_{18} solid phase extraction column was activated with 3 mL of methanol, water, and phosphate buffer, respectively. Taking the above spare liquid through the column, the flow rate was controlled to 1 drop per second. The eluent was collected, dried by nitrogen at 50 °C, dissolved with 1.0 mL of mobile phase A, filtered through 0.22 μm microporous membrane, and determined by HPLC.

Table S1 Four−parameter equation of ic−ELISA for detecting 13 FQs (ENR, CIP, SAR, OFL, NOR, PM, PEF, ENX, MAR, FLE, LOM, DAN and DIF) in PBS.

FQs	four−parameter equation
ENR	$y = 0.98979 + \frac{96.52941 - 0.98979}{(1 + (49.7258/x)^{1.03931})^{0.51187}}$
CIP	$y = 2.05185 + \frac{93.96687 - 2.05185}{(1 + (23.87916/x)^{0.9411})^{0.79843}}$
SAR	$y = 6.16339 + \frac{100.81589 - 6.16339}{(1 + (0.0589/x)^{0.49534})^{14.70523}}$
OFL	$y = 3.26284 + \frac{94.08689 - 3.26284}{(1 + (40.30906/x)^{1.2167})^{0.59914}}$
NOR	$y = 1.00423 + \frac{92.84738 - 1.00423}{(1 + (47.7086/x)^{1.49707})^{0.4363}}$
FM	$y = 4.77901 + \frac{96.73304 - 4.77901}{(1 + (19.84357/x)^{0.98489})^{1.06369}}$
PEF	$y = 3.22298 + \frac{94.99467 - 3.22298}{(1 + (15.8563/x)^{0.85774})^{1.19648}}$
ENX	$y = 4.00755 + \frac{97.55241 - 4.00755}{(1 + (1.49199/x)^{0.62693})^{4.16309}}$
MAR	$y = 3.84673 + \frac{93.82697 - 3.84673}{(1 + (1.56959/x)^{0.67014})^{4.20528}}$
FLE	$y = 1.52724 + \frac{94.27444 - 1.52724}{(1 + (13.05412/x)^{0.87524})^{1.31846}}$
LOM	$y = 2.21112 + \frac{94.06616 - 2.21112}{(1 + (22.53689/x)^{1.06598})^{0.93653}}$
DAN	$y = 4.86559 + \frac{96.03439 - 4.86559}{(1 + (14.66336/x)^{1.16851})^{1.36809}}$
DIF	$y = 1.94046 + \frac{93.11661 - 1.94046}{(1 + (0.00499/x)^{0.72887})^{295.43529}}$

Table S2 Detection limits (IC_{10}) and half maximal inhibitory concentration (IC_{50}) of ic−ELISA for detecting 13 FQs.

**name**	**IC_{10} (μg/L)**	**IC_{50} (μg/L)**
ENR	0.59	19.23
CIP	0.97	18.69
SAR	1.02	21.55
OFL	1.15	22.52
NOR	1.37	21.84
FM	1.38	20.93
PEF	1.44	21.96
ENX	1.66	21.84
MAR	1.86	21.69
FLE	2.01	21.90
LOM	2.04	22.35
DAN	2.71	20.61
DIF	3.61	22.52

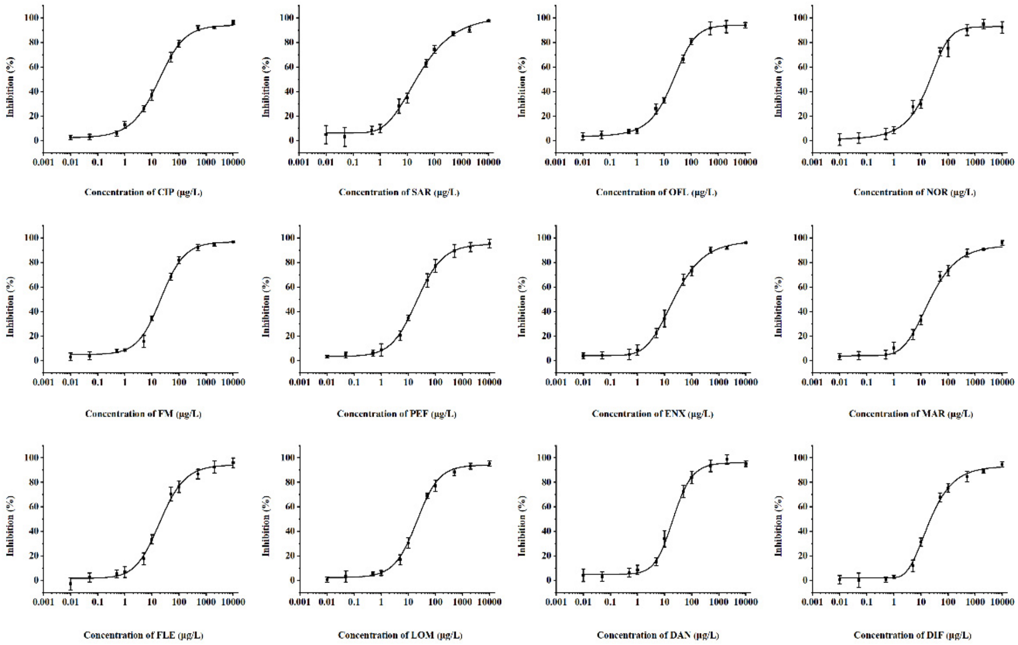

Figure S1 Standard inhibition curve of ic−ELISA for detecting 12 FQs (CIP, SAR, OFL, NOR, PM, PEF, ENX, MAR, FLE, LOM, DAN and DIF) in PBS.
